# Making Platinum Matrix for Inlay

**Published:** 1901-05-15

**Authors:** W. T. Reeves


### MAKING PLATINUM MATRIX FOR INLAY.

BY W. T. REEVES, D.D.S.

A piece of 1-1,000 platinum is cut large enough to extend well beyond all the margins other than the cervical. Anneal and place in position so that the surplus will be about equally distributed beyond the margins of the cavity; take a pledget of wet cotton or spunk and place so as to depress the platinum into the cavity, using a medium-sized, round-nosed burnisher and adding additional pledgets of the wet cotton as the platinum is carried to place, care being taken that at no time are you in danger of the burnisher going through the cotton. At this time you do not attempt to turn the platinum over the margins. Remove the cotton and matrix from the cavity, and if your packing has been thorough, you will find the platinum has been carried to every part of the cavity and your margins fairly well defined with little or no danger of breaking through the platinum. If you have broken through the platinum inside the cavity no harm will result, as the body will bridge across without danger of flowing through. If you have any surplus at the cervical margin that will press upon or hurt the gum while burnishing, trim away at this time. Anneal and return to the cavity for the second step.

Pack the cavity full of wet cotton, but not over the margins. Then, using any method that is easiest, turn the platinum over all the margins and down upon the tooth, letting the folds come where they naturally will, and fairly well burnish to the surface of the tooth. Remove from the cavity, and if the platinum has lapped over the tooth far enough to bind and hold into the cavity, trim away until the matrix will remove from the cavity freely. Anneal and return to the cavity for the third step.

The platinum that laps over on the tooth will enable you to hold the matrix in the cavity securely with your fingers. All wrinkles or folds must be burnished out and beyond the margins in all directions. It matters not if the matrix springs from one part of the cavity while burnishing upon another, although you will endeavor to hold it in place as firmly as possible; the next step will correct all faults of that nature.

A simple method by which the matrix is held firmly to every part of the cavity and at the same time will not interfere with the burnishing of every part and surface of the matrix, use a strip of rubber dam, which is drawn tightly and binds the matrix into the cavity in all directions. You can now go over all the margins and surfaces and burnish out all the spring there may be in any part of the matrix.

Now comes the task of removing the matrix from the cavity without warping it. By patient and careful teasing you can accomplish this without any warpage. If you are afraid you have sprung it ever so slightly, dry out the cavity and dry the matrix, place it in the cavity and use the rubber dam again. This time it will tease out easily. The capillary attraction of the saliva between the matrix and the tooth will often hold it very tightly.

You run more risk of warpage in the removal of any material that may be placed in the matrix to facilitate its removal from the cavity, besides the danger that some may remain upon your platinum and cause the porcelain to gas in baking. Grasp the edge of the platinum at some point as far removed from margin as possible, and where there is no fold to make it rigid, with a pair of laboratory pliers that you can lock. Burn off the saliva in the Bunsen flame to avoid any possibility of gassing. The matrix can now be handled freely in building in the porcelain, for any bending of the platinum will take place at the pliers and not near the margins.

I may have gone too much into detail, but I believe if the method is carefully followed you will have a perfect fitting matrix.—*March Dental Review.*

